# Supplementary material for: A Likelihood Approach for Real-Time Calibration of Stochastic Compartmental Epidemic Models
Source: PLoS Comput Biol. 2017 Jan 17;13(1):e1005257. doi: 10.1371/journal.pcbi.1005257 (PMC5240920; doi:10.1371/journal.pcbi.1005257)
Supplement: S1 File — (TAR.GZ) [file pcbi.1005257.s014.tar.gz › HSPH_Online-SI-Revision/Additions/IPoi10/n100-extreme-SEITR/n100-extreme-SEITR_BENCH2_table-8.pdf]

1	newly 3 weeks spec	2187.	995.102	{101., 2140.}	60.0746	0.646947	newly 3 weeks cumul	3760.	1957.34	{217., 4262.}	59.4217	0.616264	newly 5 weeks spec	7106.	2411.15	{348., 4999.}	66.0688	0.746525	newly 5 weeks cumul	14 965.	5951.73	{749., 12 605.}	61.9927	0.686504	newly 59 weeks cumul	91 886.	84 393.6	{85 688., 89 982.}	8.15402	0.192136
2	newly 3 weeks spec	265.	1.75	{0., 5.}	99.3396	2.65771	newly 3 weeks cumul	343.	4.17188	{0., 11.}	98.7837	2.3199	newly 5 weeks spec	3034.	2.75	{0., 8.}	99.9094	3.58344	newly 5 weeks cumul	4334.	8.92188	{0., 26.}	99.7941	3.11321	newly 66 weeks cumul	99 017.	20 828.9	{0., 66 806.}	78.9643	2.79521
3	newly 3 weeks spec	257.	1.78	{0., 5.}	99.3074	3.01022	newly 3 weeks cumul	379.	4.2	{0., 11.}	98.8918	2.89446	newly 5 weeks spec	2684.	5.3	{0., 16.}	99.8025	3.97474	newly 5 weeks cumul	3894.	12.71	{0., 33.}	99.6736	3.79477	newly 68 weeks cumul	98 993.	15 284.9	{0., 73 686.}	84.5596	4.4927
4	newly 3 weeks spec	698.	5.43333	{0., 16.}	99.2216	2.86609	newly 3 weeks cumul	1030.	12.2333	{0., 28.}	98.8123	2.67736	newly 5 weeks spec	3933.	11.1667	{0., 26.}	99.7161	3.5908	newly 5 weeks cumul	6733.	31.7	{0., 78.}	99.5292	3.25735	newly 66 weeks cumul	96 862.	21 744.3	{0., 71 914.}	77.5513	3.00887
5	newly 3 weeks spec	187.	1.78	{0., 5.}	99.0481	2.74739	newly 3 weeks cumul	237.	3.87	{0., 9.}	98.3671	2.50076	newly 5 weeks spec	3231.	3.71	{0., 7.}	99.8852	3.93534	newly 5 weeks cumul	4188.	10.14	{0., 24.}	99.7579	3.61208	newly 68 weeks cumul	98 880.	14 018.1	{0., 73 771.}	85.8232	4.19599
6	newly 3 weeks spec	323.	4.24675	{0., 11.}	98.6852	2.54051	newly 3 weeks cumul	460.	8.51948	{0., 22.}	98.1479	2.26717	newly 5 weeks spec	3690.	9.98701	{0., 30.}	99.7293	3.53909	newly 5 weeks cumul	5281.	23.9221	{0., 67.}	99.547	3.09854	newly 67 weeks cumul	95 041.	28 149.2	{0., 79 401.}	70.382	2.63244
7	newly 3 weeks spec	2336.	1475.17	{123., 3144.}	59.8055	0.581136	newly 3 weeks cumul	4235.	2923.72	{222., 6375.}	62.5658	0.575522	newly 5 weeks spec	6848.	3282.42	{418., 6428.}	53.8496	0.593245	newly 5 weeks cumul	15 415.	8489.91	{967., 17 477.}	55.592	0.571472	newly 59 weeks cumul	97 367.	82 552.8	{83 164., 84 894.}	15.2148	0.144988
8	newly 3 weeks spec	476.	1.25424	{0., 5.}	99.7365	3.00746	newly 3 weeks cumul	697.	4.01695	{0., 10.}	99.4237	2.58294	newly 5 weeks spec	4729.	2.42373	{0., 5.}	99.9487	3.87415	newly 5 weeks cumul	7052.	8.67797	{0., 19.}	99.8769	3.31426	newly 66 weeks cumul	99 432.	24 389.	{0., 67 103.}	75.4716	2.48617
9	newly 3 weeks spec	88.	1.21	{0., 5.}	98.625	2.5353	newly 3 weeks cumul	112.	2.96	{0., 9.}	97.3571	2.18743	newly 5 weeks spec	2227.	2.71	{0., 6.}	99.8783	3.94428	newly 5 weeks cumul	2748.	7.15	{0., 18.}	99.7398	3.44587	newly 68 weeks cumul	99 354.	13 902.6	{0., 74 933.}	86.0071	4.26619
10	newly 3 weeks spec	982.	163.551	{1., 428.}	83.3451	1.40872	newly 3 weeks cumul	1566.	309.265	{2., 772.}	80.4858	1.26236	newly 5 weeks spec	4646.	497.408	{1., 1158.}	89.2938	1.72964	newly 5 weeks cumul	8317.	1095.94	{6., 2687.}	86.8229	1.53718	newly 64 weeks cumul	99 174.	62 097.7	{20., 88 169.}	37.3851	0.758817
11	newly 3 weeks spec	578.	5.07317	{0., 13.}	99.1223	2.81538	newly 3 weeks cumul	848.	12.561	{0., 30.}	98.5188	2.4393	newly 5 weeks spec	4035.	7.4878	{0., 22.}	99.8144	3.4639	newly 5 weeks cumul	6425.	25.7805	{0., 62.}	99.5987	3.13212	newly 63 weeks cumul	99 600.	15 055.1	{0., 36 402.}	84.8844	2.94791
12	newly 3 weeks spec	936.	5.18182	{0., 14.}	99.4464	2.83266	newly 3 weeks cumul	1372.	13.0455	{1., 36.}	99.0492	2.40831	newly 5 weeks spec	5911.	9.40909	{0., 30.}	99.8408	3.5754	newly 5 weeks cumul	9781.	29.4773	{1., 76.}	99.6986	3.00737	newly 64 weeks cumul	98 924.	23 740.3	{1., 67 264.}	76.0015	2.0769
13	newly 3 weeks spec	632.	6.26923	{0., 19.}	99.008	2.66007	newly 3 weeks cumul	864.	14.1538	{0., 37.}	98.3618	2.30133	newly 5 weeks spec	4789.	12.2308	{0., 41.}	99.7446	3.41946	newly 5 weeks cumul	7468.	35.0192	{0., 104.}	99.5311	2.97812	newly 65 weeks cumul	99 507.	24 172.8	{0., 71 509.}	75.7075	2.25656
14	newly 3 weeks spec	1910.	2177.89	{332., 4468.}	75.0927	0.409458	newly 3 weeks cumul	3449.	4579.71	{648., 9780.}	90.6456	0.421609	newly 5 weeks spec	5421.	4069.56	{916., 7262.}	42.9725	0.333793	newly 5 weeks cumul	12 064.	11 708.1	{2115., 22 848.}	59.3303	0.368542	newly 58 weeks cumul	96 176.	87 173.7	{85 563., 89 074.}	9.36029	0.0427613
15	newly 3 weeks spec	1491.	633.563	{108., 1290.}	63.4851	0.585543	newly 3 weeks cumul	2425.	1319.25	{196., 2617.}	58.9158	0.506112	newly 5 weeks spec	6031.	1429.67	{219., 2692.}	76.2947	0.799982	newly 5 weeks cumul	11 560.	3709.9	{654., 7217.}	68.22	0.680819	newly 59 weeks cumul	97 278.	77 705.4	{77 116., 78 425.}	20.1203	0.0975738
16	newly 3 weeks spec	1486.	1603.35	{227., 3483.}	71.2586	0.399075	newly 3 weeks cumul	2628.	2922.56	{386., 6419.}	74.7674	0.407498	newly 5 weeks spec	4920.	4314.6	{769., 7905.}	49.3224	0.326503	newly 5 weeks cumul	10 361.	9982.	{1564., 19 871.}	58.9472	0.361053	newly 62 weeks cumul	92 728.	91 663.3	{86 491., 92 980.}	1.3135	0.00591624
17	newly 3 weeks spec	187.	1.82	{0., 5.}	99.0267	2.81236	newly 3 weeks cumul	247.	3.92	{0., 13.}	98.413	2.60737	newly 5 weeks spec	2188.	5.84	{0., 11.}	99.7331	3.84343	newly 5 weeks cumul	3077.	13.4	{0., 32.}	99.5645	3.5417	newly 68 weeks cumul	93 253.	11 730.6	{0., 67 997.}	87.4207	4.40921
18	newly 3 weeks spec	162.	2.81081	{0., 8.}	98.2649	2.3672	newly 3 weeks cumul	222.	6.36486	{0., 15.}	97.1329	1.98538	newly 5 weeks spec	2807.	6.63514	{0., 12.}	99.7636	3.50036	newly 5 weeks cumul	3654.	17.2432	{0., 35.}	99.5281	2.9266	newly 67 weeks cumul	99 640.	29 735.	{0., 78 892.}	70.1576	2.3661
19	newly 3 weeks spec	726.	4.	{0., 11.}	99.449	2.74916	newly 3 weeks cumul	1060.	10.5946	{1., 27.}	99.0005	2.30704	newly 5 weeks spec	4054.	4.32432	{0., 13.}	99.8933	3.56142	newly 5 weeks cumul	6766.	18.8378	{1., 48.}	99.7216	2.90054	newly 62 weeks cumul	99 318.	10 698.8	{1., 25 079.}	89.2278	2.24088
20	newly 3 weeks spec	300.	2.02941	{0., 7.}	99.3235	2.8586	newly 3 weeks cumul	402.	4.42647	{0., 10.}	98.8989	2.48927	newly 5 weeks spec	3692.	4.5	{0., 13.}	99.8781	3.83275	newly 5 weeks cumul	5182.	11.6029	{0., 32.}	99.7761	3.34261	newly 67 weeks cumul	98 887.	21 648.7	{0., 78 154.}	78.1077	3.24385
21	newly 3 weeks spec	120.	1.41	{0., 4.}	98.825	2.57252	newly 3 weeks cumul	157.	3.33	{0., 11.}	97.879	2.3652	newly 5 weeks spec	2417.	3.48	{0., 7.}	99.856	3.82339	newly 5 weeks cumul	3205.	9.06	{0., 27.}	99.7173	3.47178	newly 68 weeks cumul	99 034.	14 783.1	{0., 72 255.}	85.0727	4.05885
22	newly 3 weeks spec	1137.	768.267	{55., 1744.}	63.3627	0.58476	newly 3 weeks cumul	1829.	1334.87	{99., 3031.}	65.0683	0.57574	newly 5 weeks spec	5958.	2647.72	{212., 5761.}	59.1594	0.672628	newly 5 weeks cumul	10 630.	5463.37	{422., 12 024.}	59.6184	0.656953	newly 64 weeks cumul	96 878.	86 461.8	{85 019., 92 944.}	10.7519	0.233185
23	newly 3 weeks spec	1235.	71.8906	{5., 172.}	94.1789	1.56114	newly 3 weeks cumul	1958.	123.688	{10., 298.}	93.683	1.50901	newly 5 weeks spec	6598.	309.531	{19., 764.}	95.3087	1.68486	newly 5 weeks cumul	11 490.	587.078	{45., 1401.}	94.8905	1.63047	newly 65 weeks cumul	88 617.	90 480.7	{89 995., 93 138.}	5.58453	0.110043
24	newly 3 weeks spec	102.	2.24324	{0., 6.}	97.8007	2.15294	newly 3 weeks cumul	138.	4.75676	{0., 12.}	96.5531	1.8793	newly 5 weeks spec	2027.	3.2973	{0., 9.}	99.8373	3.43682	newly 5 weeks cumul	2665.	10.027	{0., 22.}	99.6238	2.93981	newly 67 weeks cumul	98 866.	23 867.3	{0., 67 280.}	75.859	2.78963
25	newly 3 weeks spec	917.	22.12	{0., 65.}	97.5878	2.34286	newly 3 weeks cumul	1410.	46.2	{1., 155.}	96.7234	2.02776	newly 5 weeks spec	3941.	52.4	{0., 127.}	98.6704	2.71304	newly 5 weeks cumul	7263.	134.32	{4., 364.}	98.1506	2.37932	newly 63 weeks cumul	94 839.	42 054.	{4., 84 466.}	55.6575	1.36967
26	newly 3 weeks spec	2138.	898.288	{55., 1788.}	67.0671	0.641533	newly 3 weeks cumul	3825.	1609.65	{105., 3232.}	68.0963	0.65383	newly 5 weeks spec	6408.	2754.55	{226., 5423.}	60.5758	0.566081	newly 5 weeks cumul	14 163.	5977.59	{452., 11 908.}	63.8072	0.602348	newly 61 weeks cumul	87 857.	92 799.1	{92 461., 93 061.}	5.62519	0.0237647
27	newly 3 weeks spec	1999.	1563.13	{317., 3738.}	63.9747	0.430726	newly 3 weeks cumul	3536.	2985.82	{602., 7275.}	67.5391	0.428356	newly 5 weeks spec	6511.	3822.05	{1104., 7890.}	49.5642	0.389242	newly 5 weeks cumul	13 890.	9355.6	{2271., 20 885.}	55.44	0.407399	newly 61 weeks cumul	98 347.	87 630.1	{85 238., 89 588.}	10.897	0.0502184
28	newly 3 weeks spec	310.	5.4	{0., 11.}	98.2581	2.78153	newly 3 weeks cumul	474.	10.46	{0., 27.}	97.7932	2.6407	newly 5 weeks spec	2971.	17.85	{0., 39.}	99.3992	3.625	newly 5 weeks cumul	4384.	37.71	{0., 85.}	99.1398	3.37748	newly 68 weeks cumul	89 828.	25 770.2	{0., 88 518.}	71.6006	3.47556
29	newly 3 weeks spec	609.	8.5098	{0., 20.}	98.6027	2.56732	newly 3 weeks cumul	951.	19.1569	{0., 50.}	97.9856	2.44698	newly 5 weeks spec	3642.	15.902	{0., 27.}	99.5634	3.36754	newly 5 weeks cumul	6162.	46.4902	{0., 107.}	99.2455	3.06337	newly 65 weeks cumul	99 052.	30 742.3	{0., 67 774.}	68.9635	2.38991
30	newly 3 weeks spec	109.	1.2	{0., 4.}	98.8991	2.60975	newly 3 weeks cumul	138.	3.18	{0., 10.}	97.6957	2.23018	newly 5 weeks spec	1649.	3.3	{0., 8.}	99.7999	3.77059	newly 5 weeks cumul	2255.	8.03	{0., 26.}	99.6439	3.29972	newly 68 weeks cumul	99 316.	14 686.1	{0., 74 935.}	85.2128	4.12732
31	newly 3 weeks spec	3137.	3890.58	{1397., 6700.}	52.4889	0.232198	newly 3 weeks cumul	6051.	7909.84	{2597., 14 277.}	59.845	0.247828	newly 5 weeks spec	6713.	7158.17	{3975., 9575.}	27.501	0.140267	newly 5 weeks cumul	17 548.	20 620.4	{9064., 32 623.}	42.1973	0.192582	newly 58 weeks cumul	87 424.	90 287.1	{87 025., 92 128.}	3.47868	0.0148001
32	newly 3 weeks spec	1418.	285.138	{30., 660.}	80.2806	0.95174	newly 3 weeks cumul	2225.	491.759	{55., 1098.}	78.709	0.90214	newly 5 weeks spec	5244.	1089.34	{117., 2693.}	79.4366	0.91904	newly 5 weeks cumul	10 289.	2144.07	{221., 5000.}	79.5222	0.921726	newly 64 weeks cumul	98 867.	91 553.5	{88 591., 93 153.}	7.39735	0.0335083
33	newly 3 weeks spec	501.	11.24	{0., 16.}	97.7565	3.06208	newly 3 weeks cumul	673.	19.16	{0., 33.}	97.153	2.99903	newly 5 weeks spec	5187.	42.64	{0., 39.}	99.1779	3.98114	newly 5 weeks cumul	7427.	82.57	{0., 88.}	98.8882	3.87434	newly 68 weeks cumul	99 003.	18 947.	{0., 84 276.}	80.8622	4.15445
34	newly 3 weeks spec	1440.	230.03	{11., 557.}	84.0257	1.12415	newly 3 weeks cumul	2363.	408.791	{21., 1081.}	82.7003	1.0839	newly 5 weeks spec	6918.	840.224	{76., 1999.}	87.8545	1.2308	newly 5 weeks cumul	12 627.	1693.9	{137., 4174.}	86.5851	1.19485	newly 64 weeks cumul	95 674.	89 960.8	{86 469., 93 101.}	5.97151	0.109362
35	newly 3 weeks spec	179.	1.78	{0., 6.}	99.0056	2.69096	newly 3 weeks cumul	229.	4.25	{0., 13.}	98.1441	2.37889	newly 5 weeks spec	2514.	5.72	{0., 13.}	99.7725	3.75576	newly 5 weeks cumul	3425.	13.29	{0., 35.}	99.612	3.34791	newly 68 weeks cumul	95 744.	15 805.	{0., 78 280.}	83.4925	3.93277
36	newly 3 weeks spec	451.	2.76316	{0., 7.}	99.3873	2.91836	newly 3 weeks cumul	672.	7.05263	{0., 16.}	98.9505	2.50645	newly 5 weeks spec	3163.	5.13158	{0., 11.}	99.8378	3.58543	newly 5 weeks cumul	5077.	16.5526	{0., 30.}	99.674	3.07503	newly 64 weeks cumul	99 501.	15 926.9	{0., 38 859.}	83.9933	2.69088
37	newly 3 weeks spec	142.	3.63793	{0., 10.}	97.4381	2.16967	newly 3 weeks cumul	189.	8.7069	{0., 18.}	95.3932	1.76841	newly 5 weeks spec	2178.	6.68966	{0., 22.}	99.6929	3.27216	newly 5 weeks cumul	2952.	20.1207	{0., 62.}	99.3184	2.74565	newly 65 weeks cumul	99 424.	27 343.1	{0., 73 442.}	72.4985	2.20235
38	newly 3 weeks spec	1570.	582.526	{99., 1318.}	67.6567	0.630892	newly 3 weeks cumul	2591.	1058.74	{197., 2375.}	65.5095	0.594592	newly 5 weeks spec	6266.	1837.58	{355., 3969.}	71.3475	0.685523	newly 5 weeks cumul	12 039.	3954.67	{746., 8703.}	69.5836	0.655691	newly 62 weeks cumul	93 543.	90 176.1	{86 187., 92 975.}	3.59936	0.016168
39	newly 3 weeks spec	955.	15.4211	{0., 50.}	98.3852	2.3671	newly 3 weeks cumul	1540.	31.0526	{1., 102.}	97.9836	2.19519	newly 5 weeks spec	4980.	36.1316	{0., 117.}	99.2745	3.04293	newly 5 weeks cumul	8707.	90.8158	{1., 303.}	98.957	2.67291	newly 64 weeks cumul	97 320.	39 630.2	{1., 78 373.}	59.2785	1.49373
40	newly 3 weeks spec	1152.	207.386	{0., 552.}	82.747	1.4061	newly 3 weeks cumul	1821.	372.456	{2., 967.}	81.142	1.33733	newly 5 weeks spec	6088.	748.	{0., 2087.}	87.7135	1.66482	newly 5 weeks cumul	10 814.	1523.19	{2., 4042.}	85.9146	1.58899	newly 65 weeks cumul	99 384.	74 008.	{2., 93 126.}	25.5333	0.755461
41	newly 3 weeks spec	84.	2.35	{0., 6.}	97.2024	2.40703	newly 3 weeks cumul	118.	4.77	{0., 12.}	95.9576	2.10675	newly 5 weeks spec	1333.	8.23	{0., 11.}	99.3826	3.53928	newly 5 weeks cumul	1781.	17.27	{0., 29.}	99.0303	3.12742	newly 68 weeks cumul	96 330.	13 776.5	{0., 71 107.}	85.6987	4.14554
42	newly 3 weeks spec	2385.	1904.	{135., 4935.}	66.2942	0.482169	newly 3 weeks cumul	4744.	3737.36	{266., 9912.}	69.3361	0.509773	newly 5 weeks spec	6151.	4172.86	{487., 8703.}	48.8673	0.397118	newly 5 weeks cumul	14 873.	10 841.3	{1026., 25 603.}	57.7586	0.445084	newly 59 weeks cumul	96 441.	91 388.5	{89 462., 92 472.}	5.23893	0.0234191
43	newly 3 weeks spec	942.	392.017	{22., 930.}	64.8968	0.735348	newly 3 weeks cumul	1522.	689.707	{41., 1658.}	63.8985	0.696646	newly 5 weeks spec	4281.	1403.93	{83., 3208.}	67.4617	0.810164	newly 5 weeks cumul	7799.	2840.1	{160., 6414.}	66.2065	0.774702	newly 64 weeks cumul	98 631.	89 107.8	{86 413., 93 101.}	9.65537	0.212213
44	newly 3 weeks spec	717.	4.29787	{0., 10.}	99.4006	2.79284	newly 3 weeks cumul	1097.	10.7021	{0., 32.}	99.0244	2.48436	newly 5 weeks spec	5449.	5.21277	{0., 12.}	99.9043	3.56032	newly 5 weeks cumul	8791.	20.617	{1., 44.}	99.7655	3.04878	newly 64 weeks cumul	99 299.	16 164.3	{1., 37 724.}	83.7216	1.99792
45	newly 3 weeks spec	514.	3.63333	{0., 8.}	99.2931	2.80792	newly 3 weeks cumul	734.	7.76667	{0., 18.}	98.9419	2.43904	newly 5 weeks spec	4552.	8.25	{0., 14.}	99.8188	3.63948	newly 5 weeks cumul	6924.	21.35	{0., 43.}	99.6917	3.15094	newly 66 weeks cumul	99 357.	24 624.9	{0., 68 328.}	75.2158	2.88421
46	newly 3 weeks spec	247.	4.04	{0., 9.}	98.3644	2.83942	newly 3 weeks cumul	335.	8.38	{0., 19.}	97.4985	2.75877	newly 5 weeks spec	2717.	10.07	{0., 12.}	99.6294	3.9247	newly 5 weeks cumul	3855.	24.9	{0., 35.}	99.3541	3.71249	newly 68 weeks cumul	99 095.	13 317.8	{0., 63 477.}	86.5606	4.36744
47	newly 3 weeks spec	977.	168.103	{17., 406.}	82.7939	1.09311	newly 3 weeks cumul	1597.	317.81	{27., 747.}	80.0995	1.01083	newly 5 weeks spec	4864.	506.845	{30., 1158.}	89.5797	1.33431	newly 5 weeks cumul	8755.	1116.98	{82., 2680.}	87.2418	1.23029	newly 63 weeks cumul	96 045.	81 014.6	{75 152., 85 301.}	15.6493	0.246414
48	newly 3 weeks spec	364.	2.32692	{0., 8.}	99.3607	2.79165	newly 3 weeks cumul	527.	6.23077	{0., 16.}	98.8177	2.48681	newly 5 weeks spec	3183.	3.86538	{0., 10.}	99.8786	3.49674	newly 5 weeks cumul	4879.	13.0577	{0., 29.}	99.7324	3.19857	newly 66 weeks cumul	97 052.	24 211.3	{0., 68 340.}	75.0532	2.68819
49	newly 3 weeks spec	1900.	2667.21	{327., 5460.}	87.3921	0.418229	newly 3 weeks cumul	3162.	4528.18	{524., 9357.}	91.123	0.407815	newly 5 weeks spec	7611.	7596.28	{1825., 12 414.}	42.2008	0.305808	newly 5 weeks cumul	14 764.	16 999.1	{3120., 31 437.}	59.4136	0.340992	newly 64 weeks cumul	95 259.	91 075.7	{87 957., 93 954.}	4.3915	0.076779
50	newly 3 weeks spec	943.	124.3	{1., 308.}	86.8187	1.38621	newly 3 weeks cumul	1533.	215.26	{3., 549.}	85.9583	1.344	newly 5 weeks spec	5024.	495.18	{0., 1219.}	90.1437	1.64215	newly 5 weeks cumul	8966.	962.84	{3., 2360.}	89.2612	1.55516	newly 65 weeks cumul	98 082.	76 783.1	{3., 93 186.}	21.7154	0.699163
